# In silico pathway analysis and tissue specific *cis*-eQTL for colorectal cancer GWAS risk variants

**DOI:** 10.1186/s12864-017-3750-2

**Published:** 2017-05-15

**Authors:** Lenora W. M. Loo, Mathieu Lemire, Loïc Le Marchand

**Affiliations:** 1 Cancer Epidemiology Program, University of Hawaii Cancer Center, Honolulu, HI USA; 2 Ontario Institute for Cancer Research, MaRS Centre, 661 University Avenue, Suite 510, Toronto, ON M5G 0A3 Canada

**Keywords:** Colorectal cancer, eQTL, Risk variant, Gene expression

## Abstract

**Background:**

Genome-wide association studies have identified 55 genetic variants associated with colorectal cancer risk to date. However, potential causal genes and pathways regulated by these risk variants remain to be characterized. Therefore, we performed gene ontology enrichment and pathway analyses to determine if there was an enrichment of genes in proximity to the colorectal cancer risk variants that could further elucidate the probable causal genes and pathways involved in colorectal cancer biology.

**Results:**

For the 65 unique genes that either contained, or were immediately neighboring up- and downstream, of these variants there was a significant enrichment for the KEGG pathway, *Pathways in Cancer* (*p*-value = 2.67 × 10^−5^) and an enrichment for multiple biological processes (FDR < 0.05), such as *cell junction organization, tissue morphogenesis, regulation of SMAD protein phosphorylation,* and *odontogenesis* identified through Gene Ontology analysis. To identify potential causal genes, we conducted a *cis*-expression quantitative trait loci (*cis*-eQTL) analysis using gene expression and genotype data from the Genotype-Tissue Expression (GTEx) Project portal in normal sigmoid (*n =* 124) and transverse (*n =* 169) colon tissue. In addition, we also did a *cis*-eQTL analysis on colorectal tumor tissue (*n =* 147) from The Cancer Genome Atlas (TCGA). We identified two risk alleles that were significant *cis*-eQTLs for *FADS2* (rs1535) and *COLCA1* and *2* (rs3802842) genes in the normal transverse colon tissue and two risk alleles that were significant *cis*-eQTLs for the *CABLES2* (rs2427308) and *LIPG* (rs7229639) genes in the normal sigmoid colon tissue, but not tumor tissue.

**Conclusions:**

Our data reaffirm the potential to identify an enrichment for biological processes and candidate causal genes based on expression profiles correlated with genetic risk alleles of colorectal cancer, however, the identification of these significant cis-eQTLs is context and tissue specific.

**Electronic supplementary material:**

The online version of this article (doi:10.1186/s12864-017-3750-2) contains supplementary material, which is available to authorized users.

## Background

According to the Centers for Disease Control and Prevention, in 2012 colorectal cancer (CRC) was the third most common cancer worldwide, with an estimated 1.4 million individuals diagnosed with CRC (www.cdc.gov). As a result, CRC is the fourth most common cause of cancer-related death, with 694,000 individuals dying from colorectal cancer annually. Risk of CRC is influenced by both environmental and genetic factors. A small percentage of CRC (3–5%) has been associated with high penetrance germline mutations, primarily contributing to the familial forms of CRC. Whereas for sporadic CRC, genome-wide association studies (GWAS) have to date identified 55 risk variants, each associated with a modest increase in risk of developing CRC.

Overall, GWAS involving thousands of cases and controls have identified hundreds of genetic variants associated with cancer or other diseases [1, 2]; however, the majority of these risk alleles are associated with a modest disease risk (OR < 1.5) [1]. One can anticipate that additional susceptibility variants will be identified through studies that have increased sample sizes, studies focusing on specific molecular sub-types, studies on other races/ethnicities, such as African Americans, Latinos and Asians, and with the application of high-throughput sequencing technologies to fine map the association signals. However, the current understanding of the biological contributions of these risk alleles has been limited. Progress has recently been made on the functional characterization of these putative causal variants through the application of tools and databases, such as expression quantitative trait loci (eQTL) analysis with the Genotype-Tissue Expression (GTEx) Project portal [3], identification of genomic modifications involved in gene regulation by the Encyclopedia of DNA Elements (ENCODE) Consortium [4], and the integration of high-resolution molecular characterization of multiple cancers by The Cancer Genome Atlas (TCGA) [5] (http://cancergenome.nih.gov/).

In this study, we conducted a Gene Ontology and pathway analysis of the genes containing, or neighboring, the 55 GWAS risk alleles for CRC in order to identify potential functional mechanisms or biological pathways that could be regulated in *cis* by these risk alleles. We also conducted a *cis*-eQTL analysis to examine the relationship between the CRC risk variants and gene expression for normal colon (sigmoid and transverse) and colorectal tumor tissue utilizing the GTEx Project portal and TCGA data, respectively.

## Methods

### Risk variants

Colorectal cancer GWAS risk variants were identified in the National Human Genome Research Institute (NHGRI) European Bioinformatics Institute (EBI) catalog of published GWAS (https://www.ebi.ac.uk/gwas/home; p ≤ 5 x 10^−8^; April 2016) [1].

### Expression quantitative trait loci analysis

The GTEx project portal (http://www.gtexportal.org/home/) was used to identify *cis*-eQTLs in normal transverse and sigmoid colon tissue for CRC risk variants (April 2016) [3]. Briefly, the cis-eQTL mapping window was defined as 1 megabase up- and downstream of the transcription start site. False discovery rate (FDR) was used for multiple hypothesis test correction [6]. Significant *cis*-eQTLs were identified based on false discovery rate FDR q-values ≤0.05. The effect size of the eQTL was defined as the slope of the linear regression—computed as the effect of the alternate allele relative to the reference allele.

We evaluated *cis*-eQTL using data from TCGA which consists of gene expression and genotype data from 155 colon adenocarcinomas and 19 normal colon tissues (from a total of 162 distinct donors: 12 matched tumor and normal adjacent pairs are included). Gene expression data was derived from an Agilent 244 K Custom Gene Expression Array and genotypes were derived from Affymetrix Genome-Wide Human SNP 6.0 Array. We used Level 3 expression data (publicly available), which consists of normalized signals and expression calls per gene, per sample. Genotype data were obtained under approved access. Gene expression levels were compared between genotypes or between normal and tumor tissues using the non-parametric Wilcoxon rank sum test when comparing two factors or Kruskal Wallis test when comparing three factors.

### Bioinformatics analysis and databases

Potential functional annotation of the risk alleles were identified using the HaploReg version 4.1database (www.broadinstitute.org/mammals/haploreg; [7, 8]). The position weight matrices modeling to examine the effects of the risk allele on transcription factor binding in HaploReg was collected from TRANSFAC [9] and JASPAR [10] databases. Transcriptional regulatory features such as DNase sensitivity, histone modifications, and transcription factor binding were examined in the University of California Santa Cruz (UCSC) Encyclopedia of DNA Elements (ENCODE) database (https://www.encodeproject.org/). The Polymorphism Phenotyping v2 (PolyPhen-2) tool was used to annotate the potential effects on protein function of coding (nonsynonymous) SNPs (http://genetics.bwh.harvard.edu/pph2/; [11]). This tool was applied to estimate the probability of the resulting amino acid substitution having a damaging effect to the protein product function. The Database for Annotation Visualization and Integrated Discovery (DAVID) v6.7 was used to identify an enrichment of biological themes, particularly Gene Ontology terms, and Kyoto Encyclopedia of Genes and Genomes (KEGG) pathways (https://david.ncifcrf.gov/home.jsp; [12, 13]).

## Results

To date, 55 risk variants have been associated with colorectal cancer in GWAS (p ≤ 5 x 10^−8^). Of these, two are coding SNPs, one synonymous (rs10936599) and one missense (rs3184504), 26 are intronic, and 27 intergenic SNPs (Table 1).

**Table 1:** Colorectal cancer risk variants

SNPs	Risk Allele	Locus	Gene(s)	Genomic position	*p*-value	OR [95%CI]	Reference
rs10911251	A	1q25.3	*LAMC1*	Intron	2 × 10^−8^	1.09 [1.06–1.12]	[45, 46]
rs6691170	T	1q41	*DUSP10 - HHIPL2*	Intergenic	1 × 10^−9^; 9.6 × 10^−10^	1.06 [1.03–1.09]; 1.06 [1.03–1.09]	[47, 48]
rs6687758	G	1q41	*DUSP10 - HHIPL2*	Intergenic	2 × 10^−9^; 2.3 × 10^−9^	1.09 [1.06–1.12]; 1.09 [1.06–1.12]	[47, 48]
rs11903757	C	2q32.3	*NABP1-SDPR*	Intergenic	4 × 10^−8^	1.16 [1.10–1.22]	[45]
rs35360328	A/T	3p22.1	*ZNF621-CTNNB1*	Intergenic	3.1 × 10^−9^	1.14 [1.09–1.19]	[49]
rs812481	G/C	3p14.1	*LRIG1*	Intron	2 × 10^−8^	1.09 [1.05–1.11]	[49]
rs10936599	C	3q26.2	*MYNN*	Synonymous	3 × 10^−8^; 3.4 × 10^−8^	1.04 [1.04–1.10]; 0.93 [0.91–0.96]	[48, 50]
rs35509282	A	4q32.2	*FSTL5 - NAF1*	Intergenic	8.2 × 10^−9^	1.53 [NR]	[51]
rs367615	?	5q21.3	*PJA2-MAN2A1*	Intergenic	4 × 10^−8^	1.35 [1.20–1.49]	[52]
rs647161	A	5q31.1	*C5orf66*	Intron	1 × 10^−10^; 4 × 10^−10^ (East Asian); 1.2 × 10^−10^	1.11 [1.08–1.15]; 1.17 [1.11–1.22] (East Asian) ; 1.11 [1.08–1.15]	[48, 53]
rs1321311	A	6p21.2	*SRSF3-CDKN1A*	Intergenic	1 × 10^−10^; 1.1 × 10^−10^	1.10 [1.07–1.13]; 1.10 [1.07–1.13]	[48, 54]
rs7758229	T	6q25.3	*SLC22A3*	Intron	8 × 10^−9^; 7.9 × 10^−9^	1.28 [1.18–1.39]; 1.28 [1.18–1.39]	[48, 55]
rs16892766	A	8q23.3	*TRPS1-EIF3H*	Intergenic	3 × 10^−18^; 3.3 × 10^−18^	1.27 [1.20–1.34]; 1.25 [1.19–1.32]	[48, 56]
rs140355816	G	8q23.3	*TRPS1-EIF3H*	Intergenic	2 × 10^−8^	1.59 [NR]	[46]
rs10505477	A	8q24.21	*FAM84B-POU5F1B*	Intron	3.2 × 10^−11^	1.17 [1.12–1.23]	[48, 57]
rs6983267	G	8q24.21	*FAM84B - POU5F1B*	ncRNA;intron	1 × 10^−11^; 7 × 10^−11^; 1 × 10^−14^	1.13 [1.09–1.18]; 1.24 [1.17–1.33]; 1.27 I[1.16–1.39]	[45, 48, 55, 56, 58]
rs7014346	A	8q24.21	*FAM84B - POU5F1B*	Intron	8.6 × 10^−26^	1.19 [1.14–1.24]	[48, 59]
rs10795668	A	10p14	*GATA3-CELF2*	Intergenic	3 × 10^−13^; 2.5 × 10^−13^	1.12 [1.10–1.16]; 0.89 [0.86–0.91]	[48, 56]
rs11255841	T	10p14	*GATA3-CELF2*	Intergenic	7 × 10^−11^	1.19 [NR}	[46]
rs704017	G	10q22.3	*RPS24-ZMIZ1*	Intergenic	2 × 10^−8^	1.10 [1.06–1.13]	[22]
rs11190164	G/A	10q24.2	*NKX2-3-SLC25A28*	Intergenic	4.0 × 10^−8^	1.09 [1.06–1.12]	[49]
rs1035209	T	10q24.2	*NKX2-3-SLC25A28*	Intergenic	5 × 10^−11^	1.12 [1.08–1.16]	[46]
rs12241008	C	10q25	*VTI1A*	Intron	1.4 × 10^−9^	1.13 [1.09–1.18]	[60]
rs11196172	A	10q25.2	*TCF7L2*	Intron	1 × 10^−12^	1.14 [1.10–1.18]	[22]
rs3824999	C	11q13.4	*POLD3*	Intron	4 × 10^−10^; 3.7 × 10^−10^	1.08 [1.05–1.10]; 0.93 [0.91–0.95]	[48, 54]
rs3802842	C	11q23.1	*COLCA2 - COLCA1*	Intron	5.8 × 10^−10^	1.11 [1.08–1.15]	[48, 61]
rs174537	G	11q12.2	*MYRF*	Intron	9 × 10^−21^	1.16 [1.12–1.19]	[22]
rs1535	A	11q12.2	*FADS2*	Intron	8 × 10^−20^	1.09 [1.04–1.13]	[22]
rs10774214	T	12p13.32	*PARP11 - CCND2*	Intergenic	3 × 10^−8^; 5 × 10^−10^	1.09 [1.06–1.13]; 1.17 [1.11–1.23]	[48, 53]
rs7136702	T	12q13.13	*LARP4 - DIP2*	Intergenic	4.0 × 10^−8^	1.06 [1.04–1.08]	[48, 50]
rs10849432	T	12p13.31	*CD9- PLEKHG6*	Intergenic	6 × 10^−10^	1.14 [1.09–1.18]	[22]
rs34245511	C	12q13.12	*LIMA1*	Intron	3 × 10^−8^	1.14 [NR}	[46]
rs11169552	C	12q13.12	*DIP2B - ATF1*	Intergenic	2 × 10^−10^; 1.9 × 10^−10^	1.09 [1.05–1.11]; 0.92 [0.90–0.95]	[48, 50]
rs3217810	T	12p13.32	*CCND2*	Intron	2 × 10^−10^	1.10 [1.06–1.14]	[45, 46]
rs10774214	T	12p13.32	*CCND2*	Intron	5 × 10^−10^	1.17 [1.11–1.23]	[53]
rs3184504	C	12q24.12	*SH2B3*	Missense	1.7 × 10^−8^	1.09 [1.06–1.12]	[49]
rs73208120	G	12q24.22	*NOS1*	Intron	2.8 × 10^−8^	1.16 [1.11–1.23]	[49]
rs4444235	C	14q22.2	*DDHD1 - BMP4*	Intergenic	8 × 10^−10^	1.11 [1.08–1.15]	[47, 48, 56]
rs1957636	A	14q22.2	*BMP4 - CDKN3*	Intergenic	1 × 10^−9^	1.08 [1.06–1.11]	[62]
rs17094983	A	14q23.1	*DACT1 - PRL31P4*	Intergenic	2.5 × 10^−10^	0.87 [0.83–0.91]	[63]
rs4779584	T	15q13.3	*SCG5 - GREM1*	Intergenic	2 × 10^−8^; 4.4 × 10 − ^14^	1.18 [1.11–1.24]; 1.26 [1.19–1.34]	[48, 64]
rs73376930	G	15q13.3	*GREM1*	Intron	1.0 10^−11^	1.246 [NR]	[46]
rs9929218	G	16q22.1	*CDH1*	Intron	1 × 10^−8^; 1.2 × 10^−8^	1.10 [1.06–1.12]; 0.91 [0.89–0.94]	[47, 48]
rs12603526	C	17p13.3	*NXN*	Intron	3 × 10^−8^	1.10 [1.06–1.14]	[22]
rs7229639	A	18q21.1	*SMAD7*	Intron	2 × 10^−8^	1.20 [1.16–1.25]	[22]
rs4939827	T	18q21.1	*SMAD7*	Intron	2 × 10^−10^; 8 × 10^−28^; 1 × 10^−12^; 1 × 10^−12^	1.12 [1.09–1.16]; 1.20 [1.16–1.24]; 1.16 [1.09–1.27] ; 0.85 [0.81–0.89]	[48, 61, 64, 65]
rs10411210	C	19q13.11	*RHPN2*	Intron	5 × 10^−9^; 4.6 × 10^−9^	1.15 [1.10–1.20]; 0.87 [0.83–0.91]	[47, 48]
rs1800469	G	19q13.2	*TGFB1- B9D2*	Intergenic	1 × 10^−8^	1.09 [1.06–1.12]	[22]
rs961253	A	20p12.3	*FERMT1 - BMP2*	Intergenic	2 × 10^−10^	1.12 [1.08–1.16]	[47, 48]
rs2423279	C	20p12.3	*BMP2 - HAO1*	Intergenic	6.64 × 10^−9^	1.10 [1.06–1.14]	[48, 53]
rs4813802	G	20p12.3	*CASC20 - BMP2*	Intergenic	7 × 10-^11^	1.10 [1.06–1.12]	[62, 64]
rs6066825	G	20q13.1	*PREX1*	Intron	4.41 × 10^−9^	1.09 [1.06–1.12]	[49]
rs4925386	C	20q13.33	*LAMA5*	Intron	2 × 10^−10^; 1.9 × 10^−10^	1.08 [1.05–1.10]; 0.93 [0.91–0.95]	[48, 50]
rs2427308	C	20q13.33	*CABLES2*	Intron	3 × 10^−11^	1.24 [NR]	[46]
rs5934683	C	Xp22.2	*GPR143 - SHROOM2*	Intergenic	7.3 × 10^−10^	1.07 [1.04–1.10]	[48, 54]

### Gene ontology enrichment analysis of genes neighboring colorectal cancer risk alleles

To determine if the 55 CRC risk variants were located in or near genes that shared similar molecular functions, or functioned in a common biological pathway, we performed gene ontology enrichment and pathway analysis of the 65 unique genes that were immediately neighboring up- and downstream of these variants. The KEGG Pathway that was significantly (FDR ≤ 0.05) enriched was *Pathways in Cancer* (*p*-value = 2.67 × 10^−5^). This pathway was enriched 6.34-fold, with 9 genes neighboring the CRC risk alleles listed in this pathway: *BMP4, CDKN1A, BMP2, LAMA5, CDH1, LAMC1, TCF7L2, TGFB1,* and *CTNNB1*. Gene Ontology analysis identified an enrichment for multiple biological processes (FDR ≤ 0.05), such as *cell junction organization, tissue morphogenesis, regulation of SMAD protein phosphorylation,* and *odontogenesis* (Table 2). The Gene Ontology molecular function category also identified *beta-catenin* (*p*-value = 1.38 × 10^−4^) and *SMAD binding* (*p*-value = 0.01); however, both were no longer statistically significant after correction for multiple-testing (FDR > 0.05).

**Table 2:** Gene Ontology Analysis - Biological Process

GO term	Genes	Fold enrichment	*p*-value	FDR
GO:0034330 ~ cell junction organization	*CD9, SHROOM2, SMAD7, LAMA5, LAMC1, TGFB1*	29.06	1.66 × 10^−6^	0.003
GO:0001763 ~ morphogenesis of a branching structure	*BMP4, BMP2, LAMA5, GREM1, TGFB1, CTNNB1*	22.38	6.09 × 10^−6^	0.010
GO:0060393 ~ regulation of pathway-restricted SMAD protein phosphorylation	*BMP4, BMP2, SMAD7, TGFB1*	64.96	2.75 × 10^−5^	0.044
GO:0002009 ~ morphogenesis of an epithelium	*BMP4, BMP2, LAMA5, GREM1, CTNNB1, NKX2-3*	16.40	2.79 × 10^−5^	0.045

### *cis*-eQTL analysis for colorectal cancer risk alleles

We also conducted a search for *cis*-eQTL (FDR ≤ 0.05) among the CRC risk alleles using data for both normal sigmoid (*n =* 124 samples) and transverse (*n =* 169 samples) colon tissue from the GTEx Project portal (Additional file 1: Table S1) and followed up significant results in colorectal tumor tissue (*n =* 155 samples) from TCGA. Of the 55 risk alleles and 65 unique genes queried in colorectal normal and tumor tissue, we identified 3 intronic risk alleles (rs1535, rs3802842, rs2427308) that were statistically significant *cis*-eQTLs for the gene containing the risk variant in normal colon tissue. We also identified two intronic risk alleles (rs34245511 and rs7229639) that were a statistically significant *cis*-eQTLs for genes neighboring the intronic SNPs, but not for the gene containing the SNP. Results are described separately for each of these risk variants below and transcriptional regulatory features [7] summarized in Additional file 2: Table S2.

#### rs1535

**Fig. 1:**
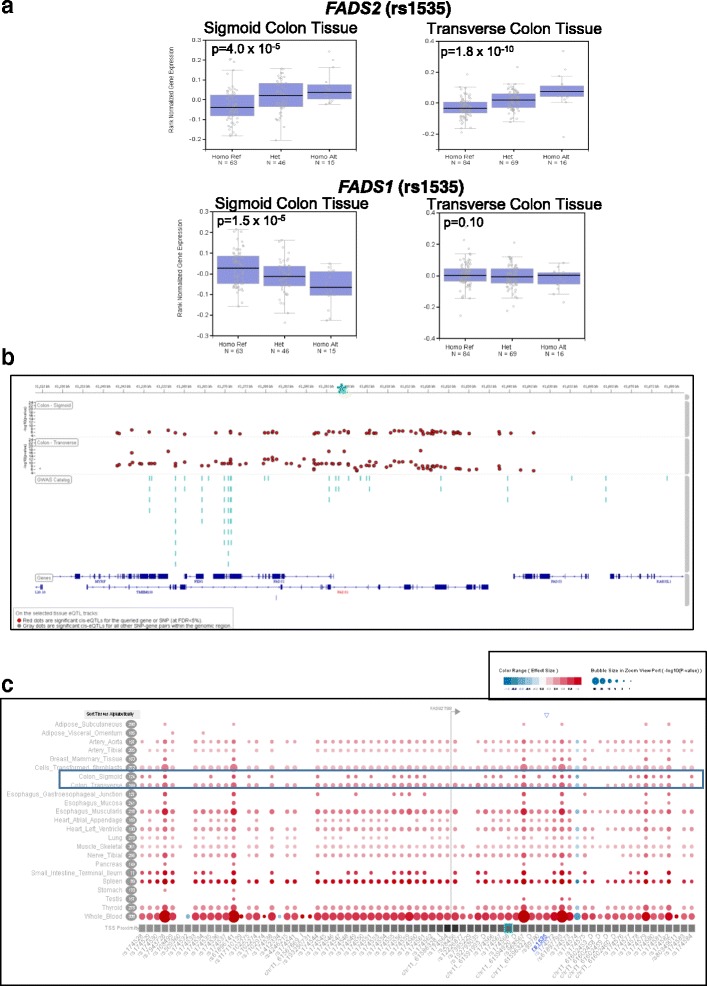
eQTL for rs1535 in Normal Sigmoid and Transverse Colon Tissue. **a** Box plots of gene expression profiles for FADS2 and FADS1 expression by genotype in normal sigmoid and transverse colon tissue. Reference Allele (risk allele) = A; Alternate Allele = G. **b** *cis*-eQTLs (+/− 1 Mb window) for FADS2 in normal sigmoid and transverse colon tissue. rs1535 is indicated by the green asterisk (*). **c** *cis*-eQTLs for FADS2 in 23 different tissue types. Bubble size represents –log10(*p*-value) and color and shading of the bubble represents effect size of the *cis*-eQTL

The CRC risk allele for rs1535 is located at 11q12. A significant *cis*-eQTL (*p* = 1.80 ×^−10^) for the fatty acid desaturase 2 (*FADS2*) gene was identified for normal transverse colon tissue. The risk allele was associated with lower expression levels of *FADS2* compared to the reference allele, with an effect size of ES = 0.36 (Fig. 1a). Interestingly, there were other multiple significant *cis*-eQTLs for the *FADS2* gene located within and in neighboring regions outside of the *FADS2* gene for normal transverse colon tissue (Fig. 1b), with rs61897795, not rs1535, being the most significant *cis*-eQTL for the *FADS2* gene (*p* = 2.40 × 10^−18^; ES = 0.64). The two SNPs have an LD of r^2^ = 0.32 (Europeans) and are located over 20 Kb apart, with rs1535 located in intron 1 and rs61897795 in intron 5 of the *FADS2* gene. In addition, other significant *cis*-eQTLs for *FADS2* were identified in multiple tissue types (Fig. 1c). The most significant is observed in whole blood for rs968567 (*p* = 7.00 × 10^−71^) with a strong effect size (ES = 1.4). rs968567 is located in the 5’-UTR of the FADS2 gene and is also a significant *cis*-eQTL in both normal transverse (*p* = 4.83 × 10^−13^; ES = 0.60) and sigmoid (*p* = 1.10 ×10^−7^; ES = 0.68) colon tissue. The protein product of the *FADS2* gene is delta-6 desaturase. It is a member of the fatty acid desaturase gene family and catalyzes the first and rate-limiting step in the biosynthesis of long chain polyunsaturated fatty acid (PUFA).

We also queried TCGA to determine if rs1535 was also a *cis*-eQTL in colorectal tumor tissue. We utilized the TCGA dataset of CRC patients (*n =* 155) with available genotype (*n =* 147) and gene expression information. Because the SNP data available for the TCGA samples did not include genotypes for rs1535, a proxy (rs174547) in high LD (r^2^ = 0.96; Europeans) was used for this analysis. We confirmed from the GTEx Project portal that it was also a significant *cis*-eQTL for *FADS2* (*p* = 1.00 × 10^−10^) in normal transverse colon tissue. We found that the proxy risk allele was associated with lower expression levels in colorectal tumor tissue compared to the reference allele (*p* = 1.5 × 10^−4^).

Available ENCODE data indicate that rs1535 is located in a region with transcriptional regulatory activity based on DNAse sensitivity and chromatin states in cell lines and in colon tissue analysis (Additional file 3: Figure S1). Histone marks (H3K4me1_Enh and H3K27ac_Enh) are detected at the locus, suggesting that this locus may function as an enhancer in the colon and other related gastrointestinal tissue. In addition, based on the 25-state chromatin model, the locus is predicted to have promoter function (4PromD2) and transcription factor chromatin immunoprecipitation sequencing (ChIP-seq) assays identified estrogen receptor alpha (ESR1) binding to the locus. Based on position weight matrix modeling, the risk allele is estimated to have a weaker affinity for ESR1 binding compared to the reference allele at rs1535.

#### rs3802842

**Fig. 2:**
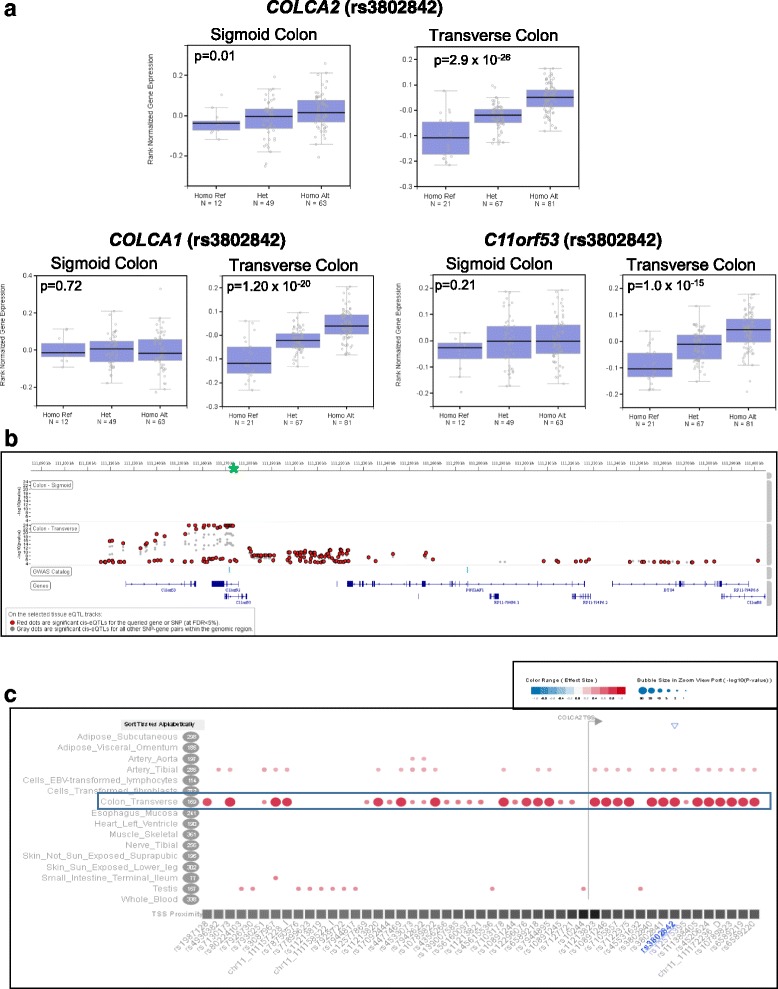
eQTL for rs3802842 in Normal Sigmoid and Transverse Colon Tissue. **a** Box plots of gene expression profiles for *COLCA2, COLCA1,* and *c11orf53*, expression by genotype in normal sigmoid and transverse colon tissue. Reference Allele (risk allele) = C; Alternate Allele = A. **b** *cis*-eQTLs (+/− 1 Mb window) for COLCA2 in normal sigmoid and transverse colon tissue. rs3802842 is indicated by the green asterisk (*). **c** *cis*-eQTLs for COLCA2 in 16 different tissue types. Bubble size represents –log10 (*p*-value) and color and shading of the bubble represents effect size of the *cis*-eQTL

The CRC risk variant, rs3802842, is located at 11q23 in the intronic regions of two overlapping genes, the colorectal cancer associated 2 and 1 (*COLCA2* and *COLCA1*) genes. rs3802842 was identified in the GTEx Project as a significant *cis*-eQTL in normal transverse colon tissue for both *COLCA2* (*p* = 2.9 × 10^−26^) and *COLCA1* (*p* = 1.2 × 10^−20^). The risk allele is associated with lower expression levels of both genes in the normal transverse colon tissue compared to the reference allele, with an effect size of ES = −0.77 and ES = −0.70 for *COLCA2* and *COLCA1*, respectively (Fig. 2a box plots). rs3802842 was also a significant *cis*-eQTL for the neighboring gene, *C11orf53* (*p* = 1.1 × 10^−15^; ES = −0.46) in normal transverse colon tissue. In addition, based on the GTEx analysis, there were multiple *cis*-eQTLs for the *COLCA2* and *COLCA1* genes in the normal transverse colon tissue (Fig. 2b). However, the risk allele, rs3802842, was not the most significant eQTL for *COLCA2, COLCA1,* and *C11orf53*. rs7130173, a SNP in high LD (r^2^ = 0.94; Europeans) with the risk allele, was the most significant *cis*-eQTL for *COLCA2* (*p* = 1.50 × 10^−26^; ES = −0.78) and therefore, may be a better candidate causal allele. The two SNPs are located over 17 Kb apart, with rs3802842 located in intron 2 of the *COLCA2* gene and rs7130173 located in intron 3 of the neighboring *C11orf53* gene. Similarly, rs3087967, a SNP in high LD (r^2^ = 0.95; Europeans) with the risk allele is the most significant *cis*-eQTL (*p* = 4.4 × 10^−21^; ES = 0.71) for *COLCA1* in normal transverse colon tissue. It is 14.8 Kb apart from the published risk allele and located in the 3’-UTR of *C11orf53*. Based on the GTEx Project data for multiple tissue types (*n =* 44), the associated effects on gene expression between rs3802842 and the three genes (*COLCA2, COLCA1*, and *C11orf53)* in the normal transverse colon tissue was specific for this tissue, it was not observed in the other 43 tissue types included in the GTEx Project. The only other significant association for rs3802842 and expression of *COLCA2* was in the tibial artery and for *COLCA1* in tibial nerve tissue (Fig. 2c).

We also did a *cis*-eQTL analysis for rs3802842 in colorectal tumor tissue using the TCGA dataset. Because the SNP data available for the TCGA samples did not include rs3802842, a proxy SNP (rs3802840) in high LD (r^2^ = 1.0; Europeans) with the risk variant was queried. The proxy risk allele, GTEx Project portal showed that it was also a significant *cis*-eQTL in normal transverse colon tissue for *COLCA2* (*p* = 2.9 × 10^−26^), *COLCA1* (*p* = 1.2 × 10^−20^), and *C11orf53* (*p* = 1.1 × 10^−15^). The *cis*-eQTL analysis with the proxy SNP demonstrated similar expression patterns in the colorectal tumor tissue samples, where the risk allele associated with lower expression levels compared to the reference allele for the *COLCA2* (*p* = 8.90 × 10^−4^) and *COLCA1* (*p* = 4.70 × 10^−3^) genes (Additional file 4: Figure S2).

rs3802842 is located in intronic regions of both *COLCA2* and *COLCA1* and based on chromatin profiling of the locus, histone marks (H3K4me1_Enh, H3K4me3_Pro, and H3K27ac_Enh) suggest transcriptional enhancer and promoter activity for this locus in normal tissue from the colon, as well as from other sites of the gastrointestinal tract. In addition, there were two SNPs (rs11213823 and rs7130173) in high LD (r^2^ > 0.8; Europeans) that also displayed promoter and enhancer histone marks as well as multiple transcription factor binding ability in ChIP seq assays. Based on chromatin marks and ChIP seq data, there is a strong indication that two SNPs - rs11213823 (r^2^ = 0.92; Europeans) located in the 5’-UTR of the *COLCA2* gene and rs7130173 (r^2^ = 0.93 Europeans) located in the intron of *C11orf53* - are located in a region of transcriptional regulatory activity. In addition, both SNPs are also significant *cis*-eQTLs for *COLCA2, COLCA1*, and *C11orf53.*

#### rs2427308

**Fig. 3:**
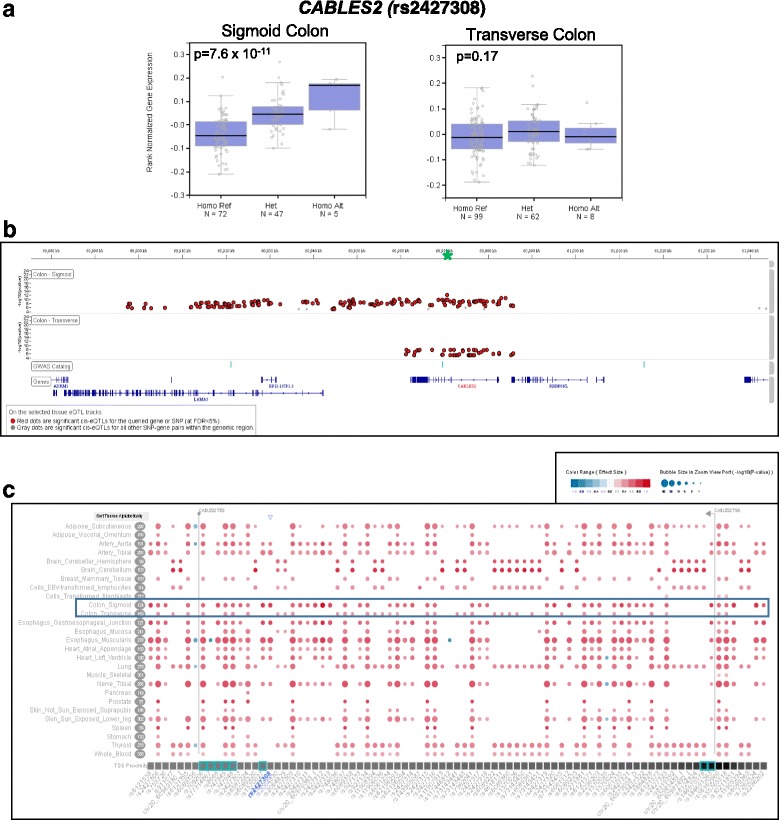
eQTL for rs2427308 in Normal Sigmoid and Transverse Colon Tissue. **a** Box plots of gene expression profiles for *CABLES2* expression by genotype in normal sigmoid and transverse colon tissue. Reference Allele (risk allele) = C; Alternate Allele = T. **b** *cis*-eQTLs (+/− 1 Mb window) for *CABLES2* in normal sigmoid and transverse colon tissue. rs2427308 is indicated by the green asterisk (*). **c** *cis*-eQTLs for *CABLES2* in 27 different tissue types. Bubble size represents –log10 (*p*-value) and color and shading of the bubble represents effect size of the *cis*-eQTL

The risk variant is located at 20q13, is a significant *cis*-eQTL (*p* = 7.60 × 10^−11^) in the normal sigmoid colon tissue for the Cdk5 and Abl enzyme substrate 2 (*CABLES2*) gene. The risk allele is associated with lower expression levels of *CABLES2* in the normal sigmoid colon tissue compared to the reference allele, with an effect size of ES = 0.76 (Fig. 3a). The risk variant is located in intron 4 of the *CABLES2* gene. *CABLES2* has multiple *cis*-eQTLs in normal sigmoid colon tissue and rs2427308 is not the most significant *cis*-eQTL for this gene. Another SNP (rs2427312) in high LD (r^2^ = 0.85; European) with risk variant rs2427308, is the most significant *cis*-eQTL (*p* = 3.8 × 10^−13^; ES = 0.93) for *CABLES2* in this tissue (Fig. 3b). The two SNPs are 1.1 Kb apart, with rs2427312 located in intron 3 of the *CABLES2* gene. Based on the GTEx analysis of multiple tissue types (*n =* 44), in addition to the sigmoid colon tissue, rs2427308 is a significant *cis*-eQTL for *CABLES2* in several tissues, including the esophagus, arteries, heart, nerve, and skin (Fig. 3c). rs2427308 is also a significant *cis*-eQTL for the neighboring genes, *LAMA5* in transformed fibroblasts and esophagus, and the *RP11-157P1.5* gene in the heart and liver. *CABLES2* has a C-terminal cyclin-box-like domain and has been shown to bind to Cdk3, Cdk5, and c-Abl [14, 15]. *CABLES2* has also been characterized as a possible tumor suppressor, functioning in both p53-mediated and p53-independent induced apoptosis pathways [16].

We also did a *cis*-eQTL analysis for rs2427308 in colorectal tumor tissue included in the TCGA dataset. Because the SNP data associated with the TCGA samples did not include rs2427308, a proxy (rs6121558) in high LD (r^2^ = 0.94) was queried. Similar to rs2427308, the GTEx Project portal showed that the proxy risk allele was also a significant *cis*-eQTL in normal sigmoid colon tissue. However, it was not a significant *cis*-eQTL in colorectal tumor tissue for the *CABLES2* (*p* = 0.45) gene. Perhaps, the high frequency of copy number alterations at the *CABLES2* gene observed in colorectal tumors contributes to the poor correlation with the *cis*-eQTL in tumor and normal tissue. Based on GISTIC analysis of the TCGA dataset [5], 65% of the tumors had a copy number gain (137 of 212) and 8% had an amplification of the *CABLES2* gene.

#### rs34245511

This risk variant is located at 12q13 in the intronic region of the *LIMA1* gene. This risk allele was not found to be a *cis*-eQTL for *LIMA1* in any of the tissue included in the GTEx Project (including normal colon tissue). However, it was identified as a significant *cis*-eQTL (FDR ≤ 0.05) for neighboring genes, such as *CERS5, COX14, LARP4, ATF1*, *ASIC1* and *DIP2B*, in other tissue types but not for normal colon tissue. Tumor tissue was not evaluated because the risk variant (or a proxy, r^2^ > 0.8) was not available for analysis in TCGA.

#### rs7229639

In addition to querying for *cis*-eQTLs for genes containing or flanking the CRC risk alleles, we queried all of the 55 CRC risk alleles for any *cis*-eQTLs (within ±1 Mb of the transcript start site) in normal sigmoid or transverse colon tissue. Of the 55 risk alleles examined, we found the risk allele rs7229639, located at 18q21 in an intron of the *SMAD7* gene, was a significant *cis*-eQTL for the lipase G, endothelial type (*LIPG)* gene (*p* = 3.7 × 10^−6^; ES = −0.58) located >636 Kb downstream of rs7229639 in the normal sigmoid colon tissue. It was not a significant *cis*-eQTL for the *SMAD7* gene in normal colon tissue or any of the tissue types (*n =* 44) included in the GTEx project. The risk allele is associated with higher expression levels of the *LIPG* gene in normal sigmoid colon tissue.

### *cis*-eQTL of missense risk variant, rs3184504

The missense risk allele, rs3184504, located at 12q24 is a coding SNP in exon 3 of the SH2B adaptor protein 3 (*SH2B3)* gene. The predicted effect of this missense mutation using the PolyPhen2 analysis tool indicates that this mutation, resulting in a Trp262Arg substitution, has a “benign” effect on protein function. In our analysis, the risk allele was not identified as a significant *cis*-eQTL for *SH2B3* in normal colon sigmoid (*p* = 0.19) or transverse (*p* = 0.47) tissue. Interestingly, it was a significant *cis*-eQTL for the *ALDH2* gene in skin tissue (*p* = 2.80 × 10^−8^), indicating that the missense risk allele in the coding region of the *SH2B3* gene has the potential to impact the expression of a neighboring gene in specific tissue types.

## Discussion

In this analysis, we evaluated the genes containing, or neighboring, the 55 CRC risk variants identified by GWAS to date for functional interactions through gene ontology enrichment and pathway analyses. The KEGG Pathway analysis revealed a six-fold enrichment for nine of the 65 genes queried are part of the *Pathways in Cancer* (*p*-value = 2.67 × 10^−5^). We also queried the GTEx Project and TCGA databases to identify *cis*-eQTLs linking the risk alleles and expression patterns of these genes. Interestingly, none of the nine genes enriched in this pathway were identified to have significant eQTLs with the risk alleles in the normal colon (sigmoid or transverse) or colorectal tumor tissue.

We also conducted eQTL analyses for each of the CRC risk variants (*n =* 55) for functional associations with genes containing the risk alleles (coding or intronic SNPs) or with immediate neighboring genes (intergenic SNPs). For each of these risk variants, or SNPs in high LD with them, we utilized normal tissue expression data available through the GTEx Project portal and colorectal tumor tissue available through TCGA to identify significant (FDR ≤ 0.05) *cis*-eQTLs in normal colon (sigmoid and transverse), followed up by the characterization of their effect in colorectal tumor tissue. We identified 4 intronic risk alleles that were significant *cis*-eQTLs in normal colon (sigmoid or transverse) tissue.

The most significant *cis*-eQTL that we identified was the risk allele, rs3802842, located at 11q23. It was associated with expression of *COLCA2, COLCA1*, and *c11orf53* in normal transverse colon tissue but not in sigmoid colon or colorectal tumor tissue. This SNP is located in the intronic region of the overlapping genes *COLCA2* and *COLCA1*. The two genes are transcribed from opposite strands, and *C11orf53* is >55 Kb upstream of rs3802842, neighboring *COLCA1*. This risk allele is associated with lower expression of all three regulated genes in both normal and tumor tissue. This *cis*-eQTL has also been previously reported by several groups for normal colon tissue [17–20] and normal rectal tissue [19]. In addition, immunohistochemical evaluation of protein expression profiles were recently reported for COLCA 1 and COLCA2 in normal colon tissue [18]. COLCA1 protein was detected in the lamina propria, extracellularly between epithelial cells, and in immune cells (eosinophils, mast cells, neutrophils, macrophages, and dendritic cells). COLCA2 is expressed in both immune and epithelial cells. Peltekova et al. observed lower levels of COLCA2 and COLCA1 protein expression in colon tissue from individuals homozygous for the risk allele, consistent with our *cis*-eQTL results, supporting the observation that the risk allele is associated with lower expression levels of *COLCA2* and *COLCA1* [18].

In addition to the risk allele (rs3802842), multiple SNPs in high LD are also significant *cis*-eQTLs for the three genes (*COLCA2, COLCA1*, and *C11orf53)* (Fig. 2b) and, as previously reported by Closa et al. 2014 [17], our analysis indicated that rs3802842 was not the most significant *cis*-eQTL in the region. In fact, rs7130173, a SNP in high LD (r^2^ = 0.94) with the risk allele had a stronger association with *COLCA2* expression and equivalent associations with *COLCA1* and *C11orf53* expression compared to rs3802842. The rs7130173 locus also demonstrated histone modification marks in normal colon tissue, suggesting that the locus has transcriptional enhancer and promoter activity [21]. However, in addition to the histone marks at the rs7130173 locus, 4 transcription factors (CTCF, SMC3, RAD21, YY1) were shown to be capable of binding to this locus based on ChIP-Seq experiments. Another SNP in high LD (r^2^ = 0.92; Europeans) with rs3802842, and located in the 5’-UTR of the *COLCA2* gene, rs11213823, also demonstrates a high likelihood of transcriptional regulatory activity, with histone marks and 6 transcription factors (CTCF, RAX5C20, RAD21, POL2, TAF1, HEY1) capable of binding to the locus and is also a significant *cis*-eQTL (*p* = 6.2 × 10^−26^) for *COLCA2* in the normal transverse colon tissue. These data suggest that either, or both, of these two SNPs could be the functional SNP regulating expression of the three genes, primarily *COLCA2.* In addition, of the 44 tissue types analyzed in the GTEx Project, these significant *cis*-eQTLs observed in the normal transverse colon tissue had a strong expression association predominantly in colon tissue and not in the other tissue types (Fig. 2c).

The *COLCA2* and *COLCA1* genes are a highly conserved, with multiple orthologs for both genes -- with 160 orthologs for *COLCA2*, from coelacanths and *Xenopus* to primates and 21 orthologs for *COLCA1*, from bats to primates. However, their function has not yet been well characterized. Since lower expression levels of *COLCA2* and *COLCA1* is associated with the risk allele (Fig. 2a) and lower expression levels of *COLCA2* and *COLCA1* were observed in colorectal tumors versus normal tissue (Additional file 4: Figure S2). It is plausible that *COLCA2* and *COLCA1* both function as a tumor suppressor gene, potentially by mediating immune cell function in the colon tissue.

The intronic risk allele, rs1535 was identified as a significant *cis*-eQTL for the *FADS2* gene in normal transverse colon tissue (*p* = 1.8 × 10^−10^; ES = 0.36) and although the association for normal sigmoid colon tissue was not statistically significant (FDR > 0.05), the strong *p*-value (*p* = 4.0 × 10^−5^; ES = 0.42) suggests that rs1535 may also be a *cis*-eQTL for sigmoid colon tissue, with lower levels of *FADS2* gene expression being associated with the risk allele. This *cis*-eQTL was also observed in colorectal tumor tissue by Zhang et al. [22]. We also observed a strong indication for association between rs1535 with FADS2 expression (*p* = 1.51 × 10^−4^) in colorectal tumor tissue; however this association did not meet the significance cut-off of FDR ≤ 0.05 after multiple text correction. Interestingly, rs1535 is also a methylation-QTL (meQTL) in lymphocytes: its genotypes, or genotypes at proxy SNPs rs174549 (r^2^ = 0.80), rs174548 (r^2^ = 0.78) and rs174537 (r^2^ = 0.94), are associated with methylation levels at various CpG sites annotated to *FADS2*, spanning its promoter region, first exon and body region [23]. Notably, the methylation levels at cg06781209, located within 1500 bp of *FADS2*’s transcription start site, increase with the number of risk alleles [23], consistent with the corresponding reduction in gene expression that we observed. *FADS2*, and a gene family member, *FADS1*, are rate-limiting enzymes that function in the biosynthesis pathway for PUFA conversion and are recognized as the main determinants of PUFA levels. rs1535 was not identified as a statistically significant *cis*-eQTL for *FADS1* in normal transverse or sigmoid colon tissue; however, the strong *p*-value (*p* = 1.5 x 10^−5^; ES = −0.39) suggests that rs1535 may also be a *cis*-eQTL for *FADS1* in normal sigmoid colon tissue. Interestingly, *FADS1* gene expression pattern associated with the risk allele is in the opposite direction compared to *FADS2*, i.e. higher levels of *FADS1* expression is associated with the risk allele (Fig. 1a). In addition, rs1535 demonstrates very poor association with *FADS1* expression in the transverse colon tissue (*p* = 0.10), the tissue type that had significant association with rs1535 and *FADS2*. These *cis*-eQTL results suggest that the effects of the rs1535 on *FADS2* and *FADS1* expression differ in the two colon subsites, with lower *FADS2* and higher *FADS1* expression levels associated with the risk allele in normal sigmoid colon tissue and robust (higher) *FADS2* and minimal change in *FADS1* expression in the transverse colon (Fig. 1a). These associated effects on gene expression could potentially result in a disruption to the relative levels of the byproducts along the long-chain PUFA biosynthesis pathway mediated by delta-6 and delta-5 desaturase. PUFAs play an important role in multiple biological systems, including the maintenance of cellular membrane lipid-bilayer integrity signaling events [24–26]. Alterations to the activity of the *FADS2* and *FADS1* gene products, delta-6 and delta-5 desaturase, respectively, has been associated with several diseases, such as diabetes, cardiovascular disease, inflammation, and cancer (breast, melanoma, lung) [27–30]. Our analysis of *FADS2* gene expression in colorectal tumor versus normal tissue indicate that *FADS2* is upregulated in colorectal tumors compared to normal tissue (*p* = 5.21 × 10^−7^) (Additional file 4: Figure S2), supporting the observation that disruption of FADS2 expression may also have an important role in CRC.

There are numerous (147) significant *cis*-eQTLs for *FADS2* in both normal transverse and sigmoid colon tissue, some *cis*-eQTLs are located in the *FADS2* gene and others in neighboring genes (Fig. 1b). The most significant *cis*-eQTL for *FADS2* in transverse colon tissue was not rs1535, it was rs61897795 (*p* = 2.40 × 10^−18^; ES = 0.64) which is in intron 5 of the *FADS2* gene. These two SNPs have a modest linkage (r^2^ = 0.32; Europeans). The linkage disequilibrium structure is quite variable across the three major racial populations at this locus (Additional file 5: Figure S3). Interestingly, the MAF for rs1535 in Europeans is 0.35, 0.57 for East Asians, and 0.11 in Africans, whereas, the MAF for rs61897795, is 0.14 for Europeans, <0.01 for East Asians, and 0.01 for Africans (frequencies based on 1000 Genomes Project Phase 3), and the association with rs1535 and CRC was initially identified in an East Asian population. If rs61897795 is identified as the functional SNP, it may have implications on the role played by *FADS2* in affecting risk of CRC among Asians and Africans compared to Europeans.

In addition to CRC, the missense risk allele, rs3184504, has been identified as a risk variant for multiple diseases (diabetes, blood pressure, coronary artery disease, arthritis, hypothyroidism, and myocardial infarction) [31–35]. This SNP was also identified as one of three pleiotropic loci with a significant association with colorectal, lung, and breast cancers [36]. rs3184504 is a missense SNP, resulting in a tryptophan to arginine substitution at position 262, the effects of this missense mutation are predicted to be benign to protein function. In addition, in our *cis*-eQTL analysis in normal and colorectal tumor tissue, we did not identify rs3184504 as a *cis*-eQTL for *SH2B3* in colon tissue or any of the other 42 tissue types included in the GTEx Project, or in colorectal tumor tissue in TCGA. However, in a separate study, Westra HJ et al. identified rs3184504 as a *cis*-eQTL for *SH2B3* and as a trans-eQTL for 14 unique genes involved in toll-like receptor and interferon-Ɣ response signaling in whole blood samples [37]. Since these cis- and trans-eQTL genes are linked to immune cell activity, the biological effects of rs3184504 could be to mediate tumorigenesis (colorectal, lung, and breast) through inflammation [38, 39].

The CRC risk variant, rs7229639, located in an intron of the *SMAD7* gene was a significant *cis*-eQTL for *LIPG* gene (*p* = 3.7 × 10–6), but not for *SMAD7* (*p* = 0.57), in the normal sigmoid colon tissue. The risk allele is associated with higher expression levels of the *LIPG* gene in normal sigmoid colon tissue. The *LIPG* gene encodes for a phospholipase involved in lipoprotein metabolism, particularly high-density lipoprotein (HDL) and vascular biology [40, 41]. A recent report by Slebe et al. demonstrated that the increased catalytic activity of LIPG is required for breast cancer cell growth and proliferation because it is responsible for hydrolyzing extracellular phospholipids, from HDL, which are then incorporated into intracellular lipids to provide the necessary lipid precursors for breast cancer cells [42]. Similarly, it is possible that the risk allele is associated with CRC by increasing levels of *LIPG* in colorectal tumor tissue to supply lipid precursors during tumorigenesis.

Our biological pathway and *cis*-eQTL analysis with the most recent list of CRC risk variants and genes in proximity to these variants, provide further insight into the potential functional relationship between the risk alleles and gene expression. However, there were limitations with our findings as regulation of gene expression is tissue and context specific, involving multiple levels of coordinated regulatory mechanisms, and the available data may have limitations in capturing these events. We utilized data from the GTEx Project portal, providing us the opportunity to examine the relationship between genotype and gene expression of 44 different tissue types from a total of 7051 samples. However, the GTEx Project only included tissue from the normal sigmoid and transverse colon. Therefore *cis*-eQTL analysis could only be performed for tissue from these colon subsites. Since only 65% of CRC originate from these two sites (23% in the distal colon and 42% in the proximal colon) [43], we may have missed identification of *cis*-eQTLs for tissue in other regions of the colon and rectum that CRC can originate in. We also did not identify statistically significant *cis*-eQTL (FDR ≤ 0.05) in the colorectal tumor tissue dataset despite a similar sample size used in the normal colon tissue analysis. We also did not observe significant *cis*-eQTLs for the three CRC risk variants (rs10795668, rs4444235, and rs9929218) that we identified in a previous report [44], perhaps due to limited sample size in the earlier report and tumor heterogeneity adding to the limitations to identify statistically significant *cis*-eQTL in tumor tissue. Copy number alterations at the loci of investigation in the tumor tissue may also complicate *cis*-eQTL identification and interpretation. This study has identified potential functional SNPs which contribute to CRC risk and tumor biology, however, additional experimental validation will be needed to confirm the role of these SNPs and genes in CRC.

## Conclusions

Our biological pathway and *cis*-eQTL analysis based on the most recent list of CRC risk variants and proximal genes provide further insight into the potential functional regulatory role for risk alleles and gene expression. The data reaffirm the potential to identify an enrichment for biological processes and candidate causal genes based on expression profiles correlated with genetic risk alleles of colorectal cancer, however, the identification of these significant *cis*-eQTLs is context and tissue specific.

## Additional files

Additional file 1: Table S1. cis-eQTL for colorectal cancer risk alleles and neighboring genes in normal sigmoid and transverse colon tissue. (XLSX 49 kb)

Additional file 2: Table S2. Transcription regulatory features of intronic colorectal cancer risk alleles and linked variants with significant eQTL of neighboring genes. (XLSX 16 kb)

Additional file 3: Figure S1. Transcriptional regulatory elements at rs1535 (chr11:61,597,490-61,598,700; hg19). ENCODE analysis of the genomic region containing rs1535 indicate a region with transcriptional regulatory activity based on DNAse sensitivity, transcription factor binding (POLR2A and ESR), and histone marks at the locus. (PPTX 111 kb)

Additional file 4: Figure S2. *Cis*-eQTL in colorectal tumor tissue and colorectal tumor versus normal tissue gene expression profiles. Box plots of gene expression profiles for *FADS2, COLCA2, COLC1,* and *CABLES2* expression by genotype (*cis*-eQTL) in colorectal tumor tissue (left column). Box plots of colorectal tumor versus normal tissue gene expression profiles for *FADS2, COLCA2, COLC1,* and *CABLES2* (right column). Indicated proxy SNPs used when SNP data available for the TCGA samples did not include genotypes of risk variants. (PPTX 170 kb)

Additional file 5: Figure S3. Linkage disequilibrium blocks of the rs1535 (11q12) locus. Linkage disequilibrium blocks (r^2^) of the genomic region (100 Kb) containing the risk variant, rs1535 (indicated by the red *) for the three major racial populations (Europeans, CEU; African, YRI; Asian, HCB). LD blocks were generated with the Genome Variation Server (GVS) tool (www.gvs.gs.washington.edu; dbSNP build 144). (PPTX 626 kb)

## Abbreviations

ChIP: Chromatin immunoprecipitation
CRC: Colorectal cancer
DAVID: Database for Annotation Visualization and Integrated Discovery
ENCODE: Encyclopedia of DNA Elements
eQTL: Expression quantitative trait loci
GTEx: Genotype-Tissue Expression
GWAS: Genome-wide association studies
KEGG: Kyoto Encyclopedia of Genes and Genomes
TCGA: The Cancer Genome Atlas
