# Supplementary figures and images for: In silico pathway analysis and tissue specific cis-eQTL for colorectal cancer GWAS risk variants
Source: BMC Genomics. 2017 May 15;18:381. doi: 10.1186/s12864-017-3750-2 (PMC5432975; doi:10.1186/s12864-017-3750-2)

## Slide 1
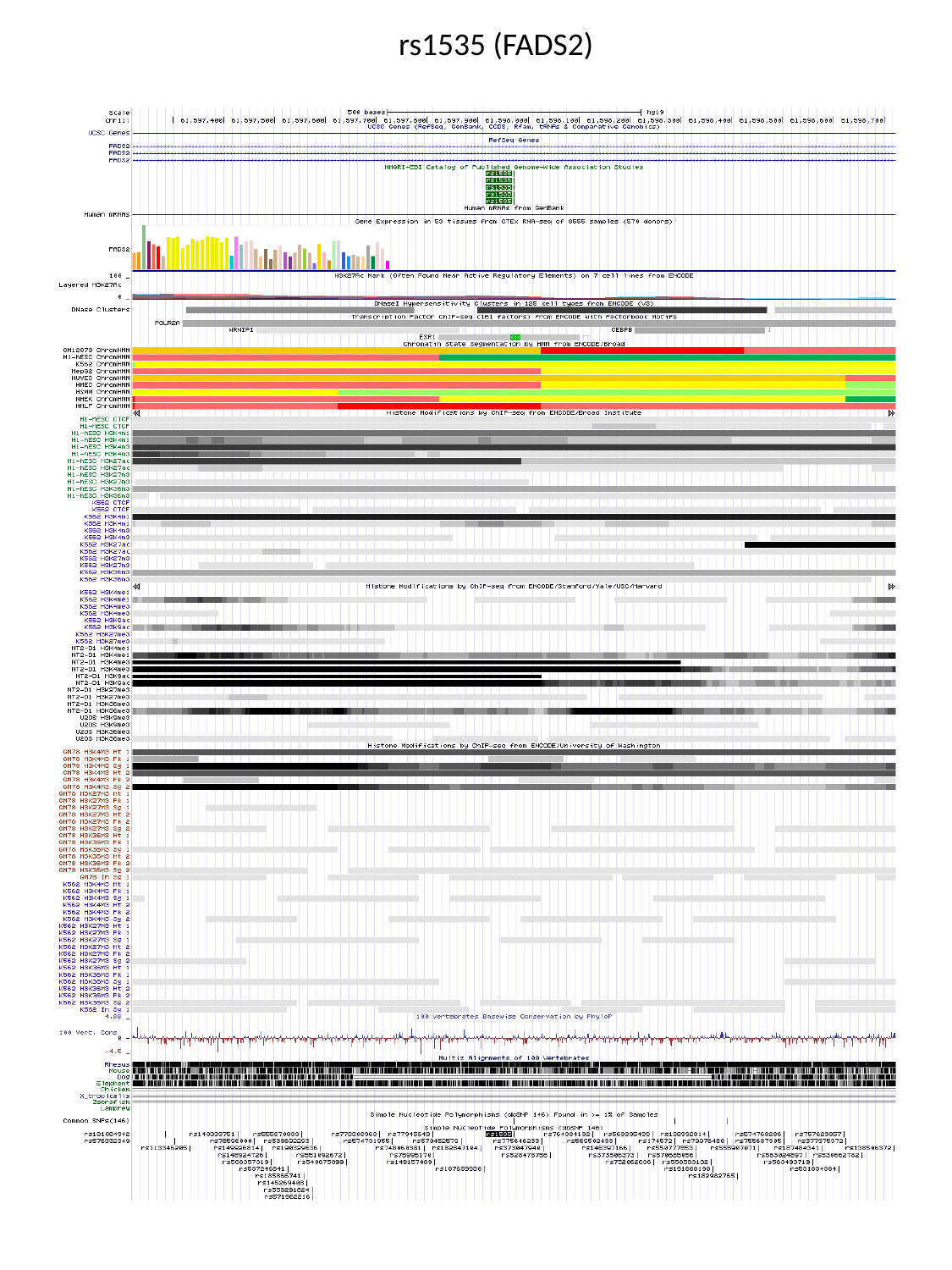

rs1535 (FADS2)

Supplement: Supplementary file 3 — Transcriptional regulatory elements at rs1535 (chr11:61,597,490-61,598,700; hg19). ENCODE analysis of the genomic region containing rs1535 indicate a region with transcriptional regulatory activity based on DNAse sensitivity, transcription factor binding (POLR2A and ESR), and histone marks at the locus. (PPTX 111 kb) [file 12864_2017_3750_MOESM3_ESM.pptx]

## Slide 1
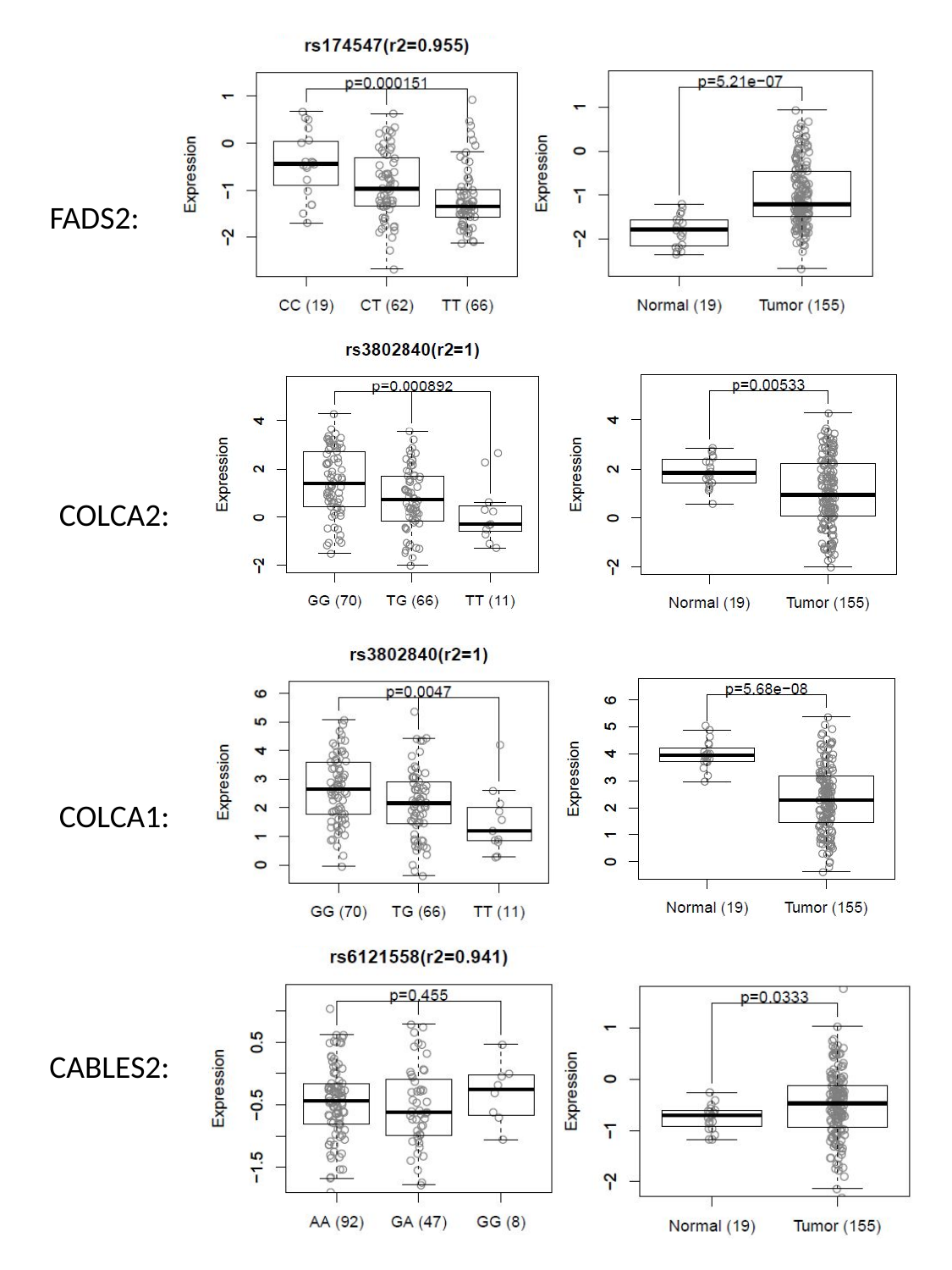

FADS2:
COLCA2:
COLCA1:
CABLES2:

Supplement: Supplementary file 4 — Cis-eQTL in colorectal tumor tissue and colorectal tumor versus normal tissue gene expression profiles. Box plots of gene expression profiles for FADS2, COLCA2, COLC1, and CABLES2 expression by genotype (cis-eQTL) in colorectal tumor tissue (left column). Box plots of colorectal tumor versus normal tissue gene expression profiles for FADS2, COLCA2, COLC1, and CABLES2 (right column). Indicated proxy SNPs used when SNP data available for the TCGA samples did not include genotypes of risk variants. (PPTX 170 kb) [file 12864_2017_3750_MOESM4_ESM.pptx]

## Slide 1
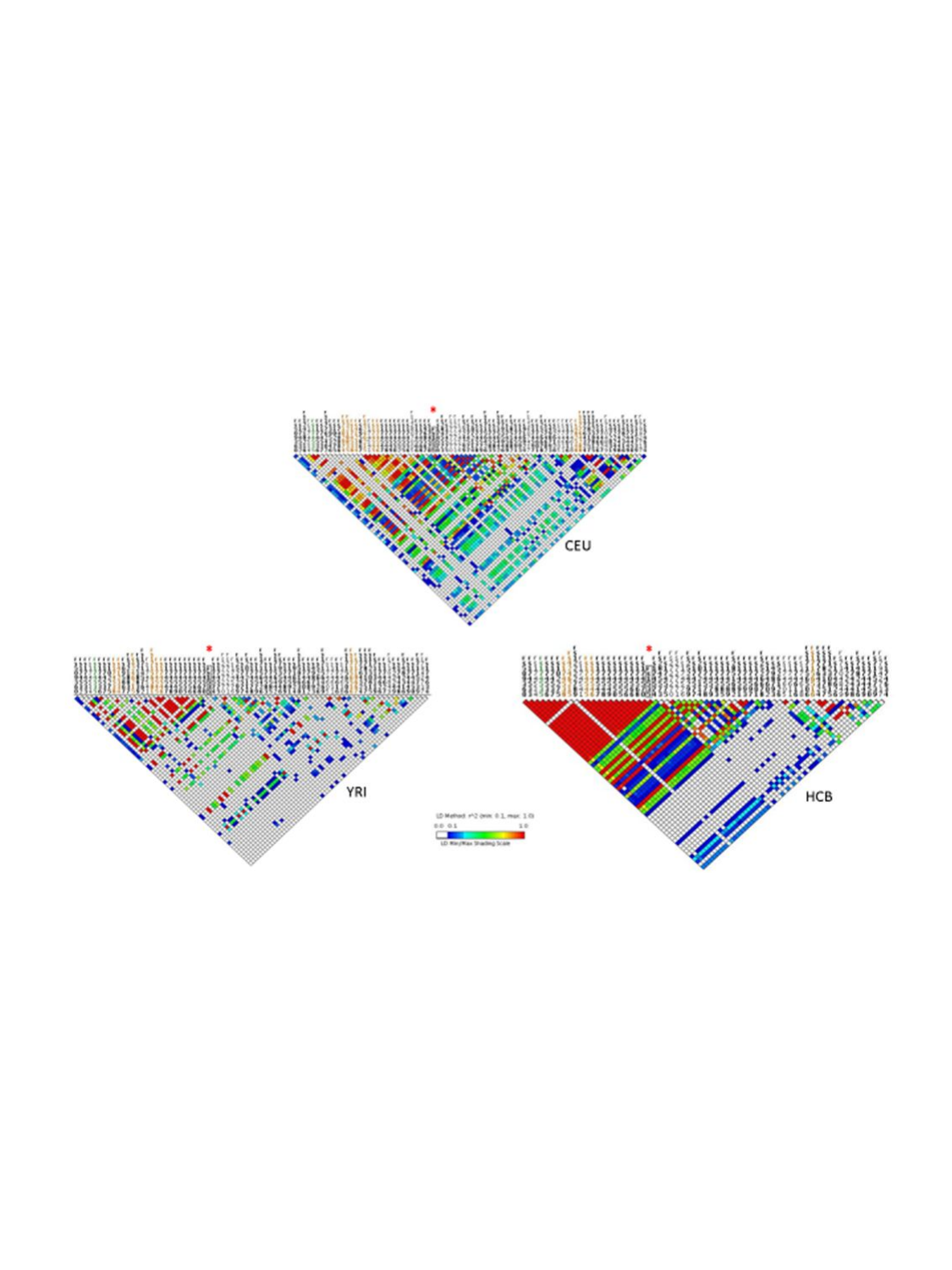

Supplement: Supplementary file 5 — Linkage disequilibrium blocks of the rs1535 (11q12) locus. Linkage disequilibrium blocks (r2) of the genomic region (100 Kb) containing the risk variant, rs1535 (indicated by the red *) for the three major racial populations (Europeans, CEU; African, YRI; Asian, HCB). LD blocks were generated with the Genome Variation Server (GVS) tool (www.gvs.gs.washington.edu; dbSNP build 144). (PPTX 626 kb) [file 12864_2017_3750_MOESM5_ESM.pptx]
